# Post-extubation use of high-flow nasal oxygenation induces upper airway leak and intrathoracic sepsis after successful Bentall procedure: A case report

**DOI:** 10.1097/MD.0000000000034240

**Published:** 2023-07-14

**Authors:** Yu-Yang Liao, Hsuan-Yin Wu, Chen-Fuh Lam, Yi-Ming Wang

**Affiliations:** a Department of Anesthesiology, E-Da Hospital and I-Shou University, Kaohsiung, Taiwan; b Division of Cardiovascular Surgery, Department of Surgery, E-Da Hospital and I-Shou University, Kaohsiung, Taiwan; c Department of Anesthesiology, Dalin Tzu Chi Hospital, Buddhist Tzu Chi Medical Foundation, Chia-Yi, Taiwan; d Department of Critical Care Medicine, E-Da Hospital and I-Shou University, Kaohsiung, Taiwan.

**Keywords:** air-leak syndrome, case report, endotracheal intubation, high-flow nasal oxygenation, postoperative, subcutaneous emphysema

## Abstract

In recent few years, high-flow nasal oxygenation (HFNO) has been widely used for management of acute hypoxemic respiratory failure and during postextubation periods, including after endotracheal intubation general anesthesia (ETGA). However, HFNO generates positive pressure in the injured airway following removal of endotracheal tube may cause airway leaks. This is the first case report of severe airway leak syndrome following postextubation use of HFNO in surgical patients.

**Patient concerns:**

This case report describes a 75-year-old female with critical aortic stenosis who underwent an emergency Bentall procedure. HFNO (flow rate of 45 L/min) was applied after weaning from mechanical ventilation and removal of the endotracheal tube.

**Diagnoses:**

At 6 hours after HFNO application, subcutaneous emphysema in the neck bilaterally and face was noted, and the emphysema extended into the supraclavicular regions.

**Interventions:**

The HFNO cannula was removed soon after and the patient was re-intubated with an endotracheal tube the following day due to progressive respiratory insufficiency. Unfortunately, the patient general condition deteriorated, as the subcutaneous air collections progressed into deep tissue infections of the neck, mediastinal abscesses, and left-sided empyema. Patient received surgical interventions repeatedly to drain the mediastinal abscess and empiric antimicrobial therapy was given.

**Outcomes:**

The patient passed away about 2 months later due to uncontrollable sepsis.

**Lessons:**

Air leaks in the upper airway can occur during the use of post-extubation HFNO use, and the resulting subcutaneous emphysema can progress to severe intrathoracic infections in surgical patients who have a sternotomy wound. Therefore, HFNO-induced subcutaneous emphysema should be treated more aggressively in open thoracic or sternotomy surgeries to prevent the development of intrathoracic sepsis.

## 1. Introduction

Specially equipped high-flow nasal oxygenation (HFNO) provides a high flow rates (up to 70 L/min) of warm and humidified mixed oxygen/gas to generate continuous positive pressure in the upper airways, wash-out of dead space in the nasopharyngeal cavity, increase alveolar recruitment and functional residual capacity, reduce work of breathing and respiratory muscle fatigue, enhance airway secretion clearance, and improve hypercapnia post endotracheal tube extubation.^[1]^ HFNO is currently used to prevent hypoxic events during anesthesia induction, procedural sedation and as an alternative to respiratory support in critically ill patients.^[1,2]^ A large-scale multi-center clinical trial further demonstrated that HFNO is not inferior to standard noninvasive mechanical ventilation for preventing reintubation and respiratory failure within 72 hours post endotracheal tube removal in high-risk critically ill adults.^[3]^ Compared with conventional oxygen therapy, HFNO also significantly reduces reintubation rates and rates of respiratory support escalation immediately after endotracheal general anesthesia (ETGA) in adult surgical patients.^[4]^ Therefore, HFNO is recommended over conventional oxygen therapy for the management of acute hypoxemic respiratory failure and for postextubation periods in emergency departments, hospital wards, intermediate or step-down units, and intensive care units.^[5]^ Although clinical studies did not find major complications in postextubation use of HFNO,^[3,6]^ positive pressure in the upper airway from high gas flow may cause air-leaks into the subcutaneous tissues from the injured airway mucosa.^[7,8]^ This case report describes a patient who was successfully weaned from invasive mechanical ventilatory support day 1 after a Bentall procedure, but developed extensive subcutaneous emphysema and pneumomediastinum within few hours after switching from endotracheal intubation to HFNO.

## 2. Case report

A 75-year-old woman with a medical history of chronic hypertension and aortic stenosis developed progressive shortness of breath and orthopnea a few days before surgery. She presented to the emergency department with hypotension and low peripheral oxygen saturation. An echocardiographic study showed calcified and severely stenotic aortic valves with a mean transvalvular pressure gradient of 60 mm Hg and left ventricle dysfunction. The proximal aortic root was dilated to a diameter of 10 cm. Acute cardiac failure was evidenced by elevated serum levels of B-type natriuretic peptide (>500 pg/mL) and pulmonary edema on chest radiography. An urgent Bentall procedure, including graft replacement of the ascending aorta and aortic valves was performed under ETGA and cardiopulmonary bypass. An endotracheal tube was inserted smoothly on the first attempt using a video laryngoscope without complications.

**Figure 1.**
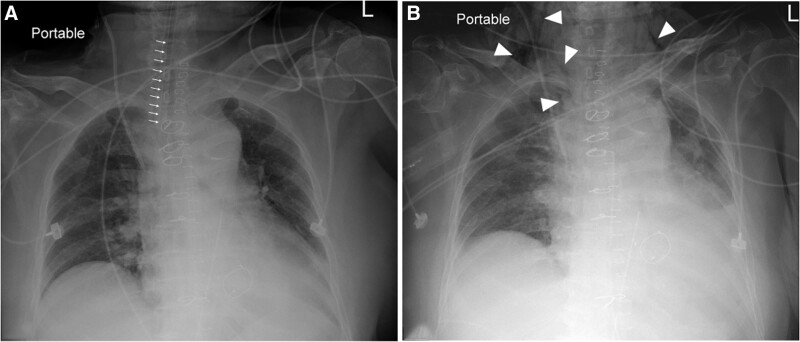
Portable chest radiographs taken on anterioposterior view. (A) Day 1 after surgery and (B) Six hours after application of high-flow nasal oxygenation following removal of endotracheal tube. Arrows indicate the position of endotracheal tube on chest radiograph of postoperative day 1. Arrowheads indicate subcutaneous emphysema in the bilateral neck and supraclavicular regions. No signs of pneumothorax or pneumomediastinum were found on these chest radiographs.

The whole surgical procedure was completed within 10 hours. After successfully weaning off cardiopulmonary bypass in the operating room, the patient was transferred to intensive care unit for postoperative care under mechanical ventilatory support. Mechanical ventilation was switched from pressure control and pressure support mode when the patient had regained consciousness and had shown effort of spontaneous respiration the morning after her operation. Following a successful spontaneous breathing trial for an hour, the endotracheal tube was removed and HFNO (Airva 2, Fisher & Paykel Healthcare, Auckland, New Zealnd) was applied with 40% fraction of inspired oxygen at a flow rate of 45 L/min via a nasal cannula (OptiflowTM; Fisher and Paykel, Auckland, New Zealand). The patient settled comfortably with HFNO, and adequate peripheral oxygen saturation was maintained. Six hours later, the patient claimed to have insidious attacks of sharp pain and swelling in the neck and face. Subcutaneous crepitus was detected at the neck bilaterally, extending to the lower jaw and the upper chest. HFNO was discontinued and the patient was put on a non-rebreathing mask for oxygen supplementation. A portable chest radiography taken at bedside confirmed the presence of subcutaneous emphysema in the neck and supraclavicular regions with no signs of pneumothorax or pneumomediastinum (Fig. 1).

**Figure 2.**
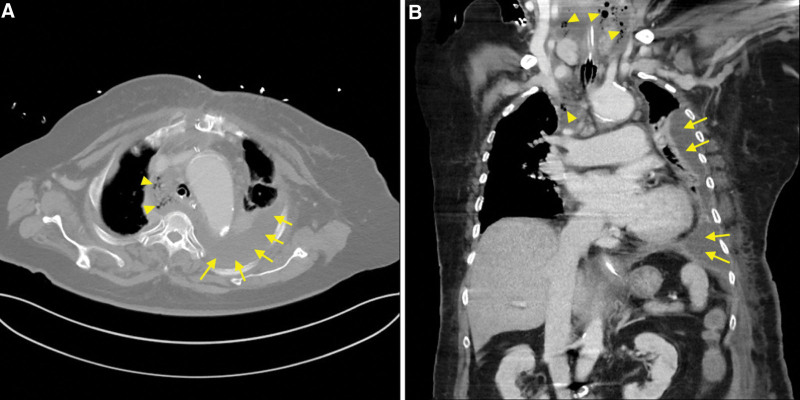
Computed tomography scans of the chest taken 6 d after detection of subcutaneous emphysema confirmed the development of empyema and mediastinal abscess. (A) Axial view and (B) Sagittal view. Arrows indicate pus collects in the left pleural cavity and mediastinum. Arrowheads indicate air locules in the subcutaneous and mediastinal regions.

At 24 hours after HFNO removal, the patient was re-intubated and mechanically ventilated due to progressive respiratory insufficiency. Over the next few days, patient developed septic shock and purulent discharge was drained from the sternotomy wound. Pseudomonas aeruginosa and acinetobacter baumannii species were isolated from the pus collections. A computer tomography of the chest found pneumomediastinum and abscess formation in the mediastinum and left pleural cavity (Fig. 2). Despite of repeated surgical interventions and empiric antimicrobial therapy to manage the intrathoracic abscesses, the patient eventually expired 2 months later due to uncontrollable sepsis and multiple organ failure.

## 3. Discussion

It is generally believed that positive airway pressure generated by HFNO in the upper airway and trachea can squeeze air into subcutaneous tissues via micro-tears in the airway mucosa.^[7,8]^ Severe air-leak syndrome was reported in 3 pediatric patients (2-month, 22-month, and 16-year-old) who were treated with HFNO after endotracheal tube removal.^[7]^ Two of these patients developed pneumothoraxes and one developed pneumomediastinum at 4 to 5 hours after HFNO use. Air leak problems in these pediatric patients were diagnosed by routine chest radiographies rather the presence of subcutaneous emphysema or hypoxic events. There was another case of an adult female with hemophagocytic lymphohistiocytosis who received endotracheal intubation and mechanical ventilatory support due to acute respiratory failure.^[8]^ HFNO therapy was applied at a flow rate of 40 L/min after weaning from mechanical ventilation. Four days later, the patient developed dyspnea and hypoxemia. A series of radiological studies were performed to investigate and found pneumothorax, subcutaneous emphysema, and massive pneumomediastinum. After HFNO cessation, the patient respiratory conditions improved. Air-leak syndrome during postoperative HFNO use after ETGA in surgical patients have not been reported in the literature.

We reported the first fatal air-leak complication following use of postextubation HFNO in a patient with a sternotomy after cardiac surgery. While most of the other case reports were associated with pneumothorax due to distal airway or lung tissue tears, the leak in our case was most likely located in the upper trachea, as the air-leak was not detected before removal of the cuffed endotracheal tube nor after tracheal re-intubation, and pneumothorax was also not found in our case. The initial clinical signs of air-leak syndrome in our patient were focal pain and detection of subcutaneous emphysema as opposed to being clinically asymptomatic or presence of hypoxia at the late stage in other cases. Therefore, detection of emphysema underneath the neck or facial subcutaneous tissues could be an early sign of upper airway leak when HFNO is applied after tracheal extubation. The outcomes of subcutaneous emphysema due to airway leak is usually self-limiting and requires mainly conservative management.^[9]^ However, our case progressed rapidly to intrathoracic sepsis and empyema due to gram negative nosocomial pathogen infections and she eventually expired because of uncontrollable sepsis. We speculated that the sternotomy wound might have precipitated the invasion of nosocomial organisms into the mediastinal subcutaneous air collection. Hence, more aggressive empirical antimicrobial treatment should be considered early stages subcutaneous emphysemas are found in surgical patients with a sternotomy or thoracotomy wound.

In conclusion, the use of postextubation HFNO is generally safe after ETGA, but air-leak syndrome may still happen in unrecognized airway mucosal injuries, as continuous positive pressure is generated by the HFNO. The presence of subcutaneous emphysema can be an early sign of airway leak following application of HFNO and subcutaneous emphysema should be treated more aggressively in open thoracic or sternotomy surgery to prevent the development of intrathoracic sepsis.

## Author contributions

**Conceptualization:** Yu-Yang Liao, Hsuan-Yin Wu, Chen-Fuh Lam, Yi-Ming Wang.

**Data curation:** Yu-Yang Liao, Yi-Ming Wang.

**Supervision:** Chen-Fuh Lam, Yi-Ming Wang.

**Validation:** Hsuan-Yin Wu, Chen-Fuh Lam.

**Writing – original draft:** Yu-Yang Liao, Hsuan-Yin Wu.

**Writing – review & editing:** Chen-Fuh Lam, Yi-Ming Wang.
